# An improved corner dealiasing and recognition algorithm for 2D Wadell roundness computation

**DOI:** 10.1038/s41598-024-60240-1

**Published:** 2024-04-24

**Authors:** Jianhuang Chen, Zhongjian Zhang, Daming Lin, Lihui Li, Wenjie Xu

**Affiliations:** 1 Department of Civil Engineering, School of Engineering and Technology, China University of Geosciences (Beijing), No. 29 Xueyuan Road, Haidian District, Beijing, 100083 China; 2 Research Institute of Highway, Ministry of Transport, Beijing, 100088 China; 3 Key Laboratory of Shale Gas and Geoengineering, Institute of Geology and Geophysics, Chinese Academy of Sciences, Beijing, 100029 China; 4 State Key Laboratory of Hydroscience and Hydraulic Engineering, Department of Hydraulic Engineering, Tsinghua University, Beijing, 100084 China

**Keywords:** Wadell roundness, Corners recognition, Digital image processing, Outline dealiasing, Cyclic midpoint filtering, Petrology, Civil engineering

## Abstract

This paper optimizes the 2D Wadell roundness calculation of particles based on digital image processing methods. An algorithm for grouping corner key points is proposed to distinguish each independent corner. Additionally, the cyclic midpoint filtering method is introduced for corner dealiasing, aiming to mitigate aliasing issues effectively. The relationships between the number of corner pixels (*m*), the central angle of the corner (*α*) and the parameter of the dealiasing degree (*n*) are established. The Krumbein chart and a sandstone thin section image were used as examples to calculate the 2D Wadell roundness. A set of regular shapes is calculated, and the error of this method is discussed. When *α* ≥ 30°, the maximum error of Wadell roundness for regular shapes is 5.21%; when 12° ≤ *α* < 30°, the maximum error increases. By applying interpolation to increase the corner pixels to the minimum number (*m*_*0*_) within the allowable range of error, based on the *α*-*m*_*0*_ relational expression obtained in this study, the error of the corner circle can be minimized. The results indicate that as the value of *m* increases, the optimal range interval for *n* also widens. Additionally, a higher value of *α* leads to a lower dependence on *m*. The study's results can be applied to dealiasing and shape analysis of complex closed contours.

## Introduction

The size and shape of granular materials have an impact on many engineering and technical fields^1–3^. For example, in the field of hydraulic fracturing research^4,5^, specialized hydraulic fracturing sand has strict requirements for the size and shape of particles, and its ideal shape is a perfect spherical shape without sharp corners^6^. The shape of particles has a significant effect on interactions with fluids^7^. In the field of macro- and microgeotechnical mechanics, the shape of particles can affect the mechanical properties of sand^8,9^. Researchers have conducted the discrete element method (DEM) simulations to study the macroscopic and microscopic mechanical behavior of sand particles^10–15^, based on the true shape of the particles and the generation of angular particles^16–18^.

**Figure 1:**
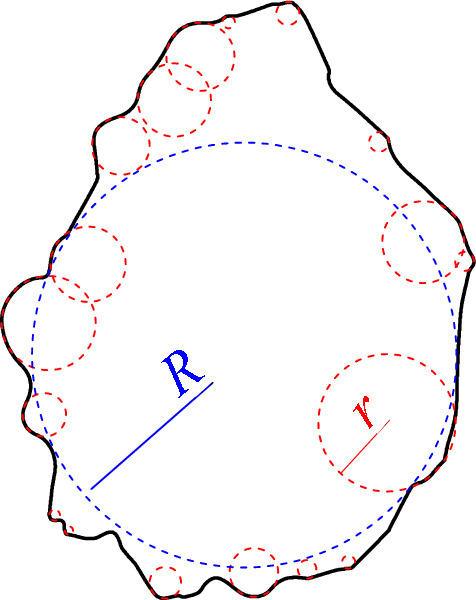
2D Wadell roundness diagram.

The study of particle shape can be traced back to the beginning of the last century. Researchers have conducted quantitative research on the shape of rock and soil particles and proposed a series of parameters to describe particle shape^19,20^, such as sphericity^21,22^, convexity^23^, aspect ratio^24^, and Wadell roundness^21,22,25^. Among them, Wadell roundness is an indicator used to describe the relative sharpness of particle corners. 2D Wadell roundness is expressed by the ratio of the mean curvature radius of each corner of the particle to the maximum inscribed circle (MIC) radius^21^, as shown in Fig. 1. The specific calculation equation is as follows:

1 $$\Pi =\frac{{\sum }_{i=1}^{N}{r}_{i}}{N\cdot R}$$

where *r*_*i*_ is the curvature radius of the ith corner of the particle, *R* is the MIC radius of the particle, and *N* is the number of corners of the particle.

When computer hardware performance has not yet been developed, calculating 2D Wadell roundness requires comparing the curvature radius of each corner of the particle with that of a circular hole ruler, and the measurement method is very inefficient. Therefore, Krumbein & Sloss created a chart for quickly determining the sphericity and Wadell roundness levels of particles^22,26^. At present, digital image processing and 3D scanning methods can be used to obtain 2D and 3D outline information of particles for Wadell roundness calculation^25,27^, and corner circles or balls can be fitted at the corners to estimate the curvature radius. The greatest advantages of digital image processing are its low cost, easy acquisition, and small data volume. The obtained particle outline is representative, and extremely fine particles (such as petrographic thin-sections) can be imaged through a microscope^28^. Furthermore, recent studies have demonstrated a strong, and in some cases linear, correlation between the 2D and 3D morphology characterization parameters of particles^29–31^. This correlation suggests the potential for converting between 2D and 3D morphological parameters, although such conversions should be made with careful consideration of the underlying assumptions.

The advantage of Wadell roundness is that it can fully consider the impact of each corner, but the disadvantage is that it is highly sensitive to the jagged edges around the particle outline, which affects the recognition of the particle corner position and the calculation of the curvature radius^32^. Because the essence of the image is a pixel matrix, the particle outline obtained from the image inevitably has jagged edges, which is a small range of random errors. Random errors often do not have clear directionality and fluctuate near true values. However, the calculation of the curvature radius depends on the overall smoothness of the identified scale, and the presence of a sawtooth leads to significant fluctuations in the curvature. If the sawtooth is not removed, it is possible to recognize the edge as a particle corner.

For the above reasons, a holistic denoising method is currently used to obtain smooth particle outlines. Zheng used a locally weighted average combined with the k-fold cross-validation method to denoise the particle outline, and the denoising standards were relatively objective^33^. Nie used a closed B-spline curve function to reconstruct the outlines of particles for dealiasing. The combination of high-degree curve fitting corners and low-degree curve fitting noncorner parts has some pertinence^34^. Vangla used the image fast Fourier transform and linear polygon approximation to denoise the particle outline, and the smoothing effect was significant^32^. Another method is to convert the particle outline into the frequency domain through the Fourier transform, thereby obtaining a smooth particle outline^35^. In this method, any particle outline can be composed of sine and cosine waves, which are included in the category of parameter fitting. This method can obtain a fitting equation for the particle outline, vectorize the particle outline, and achieve reconstruction similar to that of particles^17,36^.

However, it is difficult to balance the smoothness and nondeformation of the particle outline during dealiasing, as this requires a compromise between insufficient and excessive^20,37^. When the dealiasing is insufficient, the particle outline will still have jagged edges, while excessive information will cause outline deformation. The current methods for calculating Wadell roundness based on digital image processing cannot accurately determine the degree of dealiasing or do not take the differences between corners into account, resulting in inaccurate recognition of the corner position and calculation of the curvature. The method of converting particle outlines to the frequency domain has low computational efficiency when fitting details, and it is difficult to fit sharp corners, resulting in generally blunt results.

Therefore, this paper proposes a nonparametric filtering method called the cyclic midpoint filtering method based on the equidistant and closed-loop characteristics of particle outline points, with a focus on studying the degree of dealiasing of particle corners. The optimal range of parameters for controlling the degree of dealiasing was calibrated to ensure a smooth particle outline while reducing deformation at the corners. A grouping algorithm was proposed to distinguish each independent corner point. The algorithm combines dichotomy and cyclic methods, making it more effective for corner recognition. Relevant researchers can utilize the proposed methods to achieve reliable calculations of 2D Wadell roundness.

## Methods

### Outline extraction

The image topological structure analysis algorithm proposed by Satoshi Suzuki in 1985 is used to extract the outlines of binary image particles^38^.

### Outline dealiasing

**Figure 2:**
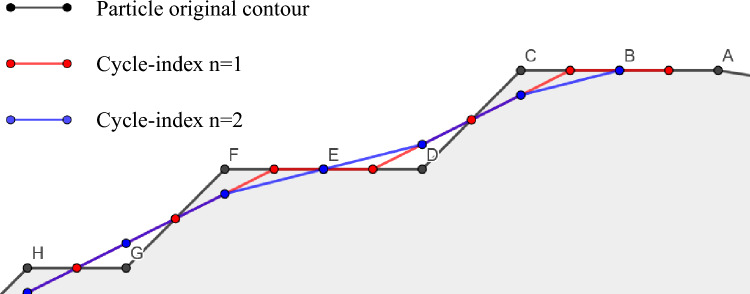
Schematic diagram of the cyclic midpoint filtering method.

This section proposes a nonparametric filtering method for particle outline dealiasing, which we call the cyclic midpoint filtering method. The characteristic of nonparametric filtering methods is that they do not need to find a function model to fit the patterns of the data. The principle of the cyclic midpoint filtering method is to gradually apply the midpoint of existing particle outline points as a new outline and use it as a loop. The specific operation of this method is as follows: The particle outline points are connected sequentially, and the midpoint of each line segment is taken as the new outline point. The outline with the number of cyclic for dealiasing degree *n* = 1 (red line) is obtained by connecting the midpoint of the original outline points of the particle. Based on this, the midpoint is connected to obtain an outline with *n* = 2 (blue line), as shown in Fig. 2. By repeating this operation, the effect of gradually removing aliasing from the particle outline is achieved. When using this method, the number of cycles is adjusted as needed to achieve different smoothing effects.

### Corner key point recognition

(1) The cyclic midpoint filtering method is utilized to perform preliminary overall noise reduction on the particle outline. The accuracy requirement for noise reduction is not high in this step, as the purpose is only to identify corners rather than calculating the curvature radius. Based on the number of particle outline points, the number of cycles is preliminarily determined, and the overall noise reduction can be controlled to basically no sawtooth edges. (2) Calculate the MIC of the particle outline. The particle image is discretized into pixels, and the center of the MIC is the point farthest from the outline of the particle interior. According to this idea, each pixel inside the particle outline is calculated as the nearest distance between the pixel and the boundary. The pixel point corresponding to the maximum distance is the center of the MIC^39^. (3) Search for corner key points on the particle outline. Three adjacent points on the after dealiasing particle outline are taken to create a circle. If the center of the circle is inside the outline, the radius is less than the MIC, and the circle does not exceed the particle outline after dealiasing; then, the middle of these three points are marked as corner keys. All outline points that meet the above conditions are the set of particle corner key points.

### Corner key point grouping

**Figure 3:**
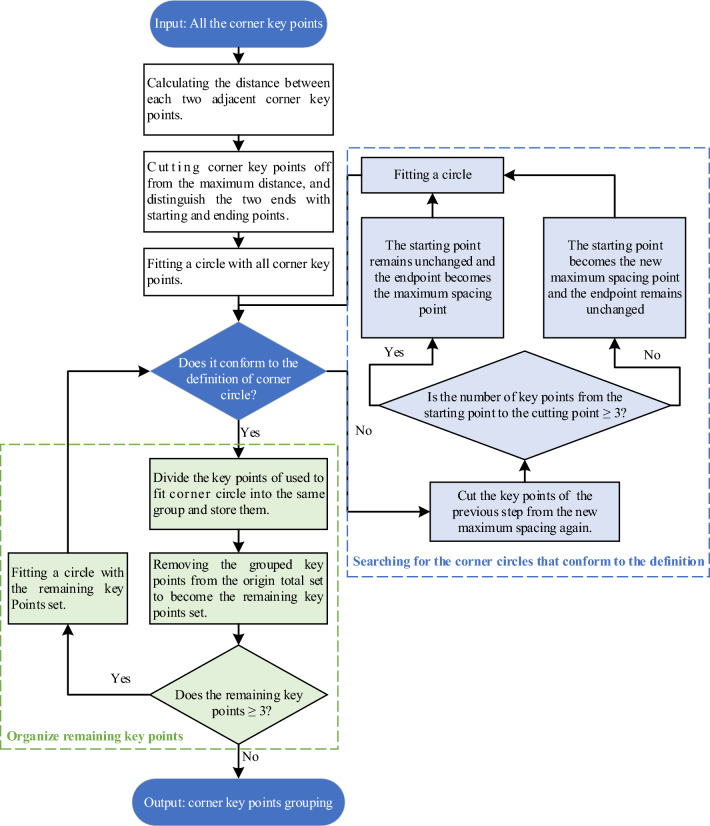
Corner key points grouping process.

We have introduced an innovative algorithm that combines dichotomy and cyclic methods to group corner keypoints to distinguish each individual corner. The key points are grouped according to the different corresponding corners. Therefore, we divided the grouping strategy into three steps: (1) Cut off the key points from the maximum spacing and distinguish the two ends with starting and ending points. If the particle has more than one corner, the group gap is most likely at the maximum spacing of these key points. (2) Corner key points are fitted to circles, and whether they satisfy the definition of the corner circle is determined^22,40^. According to the definition of 2D Wadell roundness, the radius of the corner circle should be smaller than the MIC radius, and the corner circle should be tangent to the particle outline. Fitting a circle starts with all the corner key points; if it does not conform to the definition of the corner circle, the key points used to fit the circle will be cut off again from the next maximum spacing to fit a circle again. Before fitting a circle, it is necessary to determine whether the number of key points from the starting point to the cutting point is greater than or equal to 3. If so, the starting point remains unchanged, and the endpoint becomes the maximum spacing point. Otherwise, the starting point becomes the new maximum spacing point, and the endpoint remains unchanged. The above operation is repeated until a defined corner circle is fitted. If the fitting circle conforms to the definition of the corner circle, the key points used to fit the corner circle are grouped together and removed from the complete key points set. (3) A circle is fitted with the remaining corner key points, and whether the circle conforms to the definition of a corner circle is determined. The judgment method is consistent with the second step until the remaining corner key points do not exceed 3, and the grouping of corner key points is output. The specific process of grouping corner key points is shown in Fig. 3.

### Calculation of the curvature radius and circle fitting

**Figure 4:**
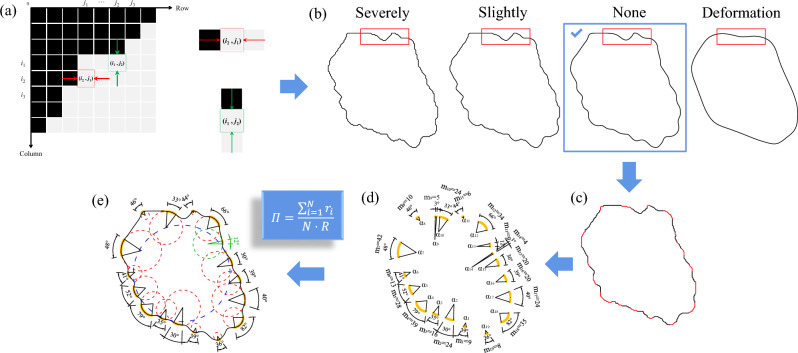
Wadell roundness calculation process. (**a**) Particle outline extraction. (**b**) Particle outline dealiasing. (**c**) Corner recognition. (**d**) Corner dealiasing. (**e**) Particle corner circle fitting. The innovation points of this paper are in parts (**b**–**d**).

(1) The cyclic midpoint filtering method is used to remove sawteeth from the original outline for particle corners. Regular graphics with known 2D Wadell roundness theory values, central angle of corner (*α*) and export images with different resolutions are designed. The relationships between the number of pixels at the corner (*m*) and between *α* and the number of cyclic filters (*n*) are established. The optimal cycle number range is calibrated for different *α* and *m* values to balance the smoothness and nondeformation of the corners. The specific calibration process is discussed in Section “Verify the calculation results using regular shapes” of this article. During the dealiasing process, the corners that achieve the best noise reduction are extracted at any time to obtain fine corners, as shown in Fig. 4d. (2) Use the after dealiasing outlines of the corners obtained from step 1 to fit the corner circle by using the least squares method^40^. However, when *α* is too small, as shown by the green dashed circle in Fig. 4e, the corner circle does not correspond to the particle's corner. Larger corners and fewer key points contradict each other. This issue objectively exists and is not caused by the methods used in this study. There is no clear value for how small an *α* is to be considered a corner. Based on our experience in the calculation process, it is assumed that when *α* < 12°, the corner circle does not correspond to the particle corner.

 According to Eq. (1), the 2D Wadell roundness is calculated by dividing the mean curvature radius of all corners (red dotted circle) of the particle by the radius of the MIC (blue dotted circle). The entire calculation process is shown in Fig. 4.

## Results

### Verification of the calculation results in the Krumbein chart

**Figure 5:**
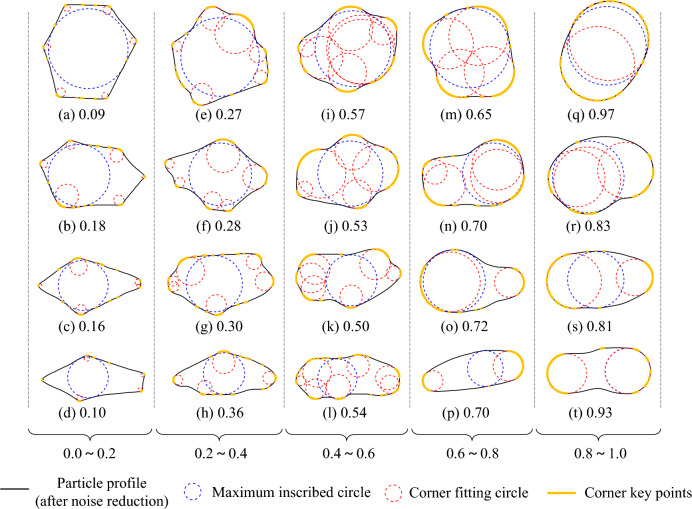
The 2D Wadell roundness calculation results and the Krumbein chart reference range.

In this section, the 2D Wadell roundness of 20 particles in the Krumbein chart^22^ is calculated using the method presented in this article. The roundness values, corner key points, MIC, and corner circles of these particles were obtained, as shown in Fig. 5. The corner key points algorithm identifies potential corner regions. After grouping and fitting the circles with corner key points, each corner circle is tangent to the particle outline, which conforms to the definition of corner circles. The calculation results are consistent with the reference range provided by Krumbein & Sloss.

### Calculation of Wadell roundness of particle minerals in a petrographic thin-section image

Observation of petrographic thin sections is a low-cost method for effectively obtaining information on the microstructure of rocks. In the field of oil and gas, the roundness of mineral particles is one of the important results of thin-section identification. According to the standardized process for thin-section identification, technicians are required to classify the roundness of mineral particles by comparing them with Krumbein charts or similar diagrams. The roundness is divided into five levels: angular (0–0.15), subangular (0.15–0.25), subcircular (0.25–0.4), circular (0.4–0.6), and extremely circular (0.6–1.0).

**Figure 6:**
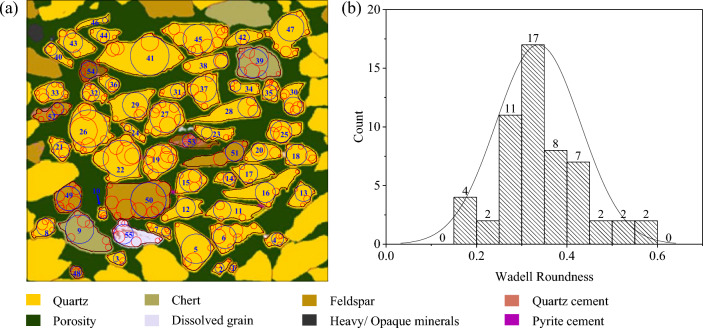
Calculation and statistical results of corner circles in mineral particle images of a sandstone thin section. (**a**) Image of sandstone mineral particles from Das^41^ and calculation results of the particle corner circles; (**b**) Statistical results of the particle roundness distribution.

An image is cited as an example in this section. This image is a sandstone slice from a deep-water oil field in Mexico that has undergone image particle segmentation^41^. The calculation result of the corner circle of roundness is superimposed on the original image, as shown in Fig. 6a. These mineral particles are mainly composed of subcircular, with 36 particles accounting for approximately 65.5% of the total, as shown in Fig. 6b. The roundness of 55 mineral particles was calculated according to the different mineral types, as shown in Table 1. Although the mineral types are different, the particle roundness values are very close, and the average roundness of all particles is also subcircular.

**Table 1:** Average Wadell roundnesse of the different mineral types.

Mineral types	Quartz	Chert	Feldspar	Dissolved grain	Total average
Wadell roundness	0.34	0.30	0.34	0.34	0.34

## Discussion

### Verify the calculation results using regular shapes

**Figure 7:**
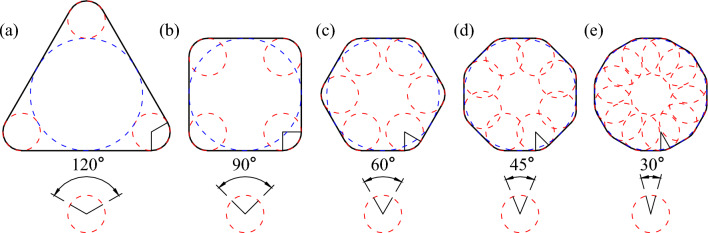
Regular graphs with known theoretical roundness values. (The theoretical roundness is 1/3).

This section designs a set of regular graphs with known 2D Wadell roundness theory values to test the method proposed in this paper. First, the MIC and corner circles of these figures are the same, and all the 2D Wadell roundness theory values are 1/3. Second, the *α* values of these figures are different, gradually decreasing from 120° to 30°, as shown in Fig. 7. Finally, these graphics are exported into images at different resolutions, and 2D Wadell roundness is calculated, as shown in Table 2.

**Table 2:** Picture information of the regular graphics and 2D Wadell roundness calculation results.

*α*	*m*	*R*	Π	Error (%)	Optimal number of cycles (*n*_*0*_)	Optimization range (3% error range)
120°	9	14.32	0.33373	0.12	16	13 ~ 18
	43	64.40	0.33359	0.08	16	4 ~ 37
	58	89.07	0.33104	0.69	21	5 ~ 41
	87	134.61	0.32916	1.25	24	4 ~ 63
	115	179.98	0.33323	0.03	20	5 ~ 98
90°	10	19.00	0.33605	0.81	6	4 ~ 6
	19	40.00	0.33305	0.08	7	6 ~ 9
	29	59.00	0.33295	0.11	16	2 ~ 26
	47	100.00	0.33355	0.07	23	2 ~ 48
	68	140.00	0.33326	0.02	43	4 ~ 106
	86	180.00	0.33338	0.01	41	4 ~ 112
60°	19	57.98	0.33507	0.52	8	6 ~ 11
	30	96.90	0.33348	0.04	25	16 ~ 32
	42	136.69	0.33361	0.08	25	13 ~ 44
	55	179.74	0.33336	0.01	34	15 ~ 77
45°	32	132.48	0.34113	2.34	24	24 ~ 32
	42	179.61	0.34045	2.13	19	9 ~ 28
	51	215.59	0.33808	1.43	22	7 ~ 84
	64	270.29	0.33936	1.81	40	8 ~ 108
	78	327.82	0.33329	0.01	39	9 ~ 226
30°	59	351.06	0.32371	2.90	20	18 ~ 21
	74	441.11	0.33418	0.25	5	2 ~ 8
	90	537.16	0.35070	5.21	6	–
	108	645.22	0.34860	4.58	8	–

(1) *α* is the central angle of the corner, *m* is the number of pixels at the corners, *R* is the maximum inscribed circle radius of the particle, and Π is the value of 2D Wadell roundness. (2) "–" means that the error exceeds 3%. (3) The theoretical roundness value is 1/3.

When *α* is the same, corners with different degrees of sharpness can be considered this type of scaling. For example, for multiple corners with *α* = 120° on a particle, even though their sharpness varies, the same type of scaling can be considered, with the difference being the number of pixels at the corners. Therefore, the regular graph shown in Fig. 7 can be seen as a uniform and relatively broad coverage of the possible angular situations encountered in the 2D Wadell roundness calculation process. Table 2 lists the 2D Wadell roundness calculation results and corresponding errors. The two highest errors of 5.21% and 4.58% both occur at *α* = 30°.

### Discussion on the degree of dealiasing for corners

**Figure 8:**
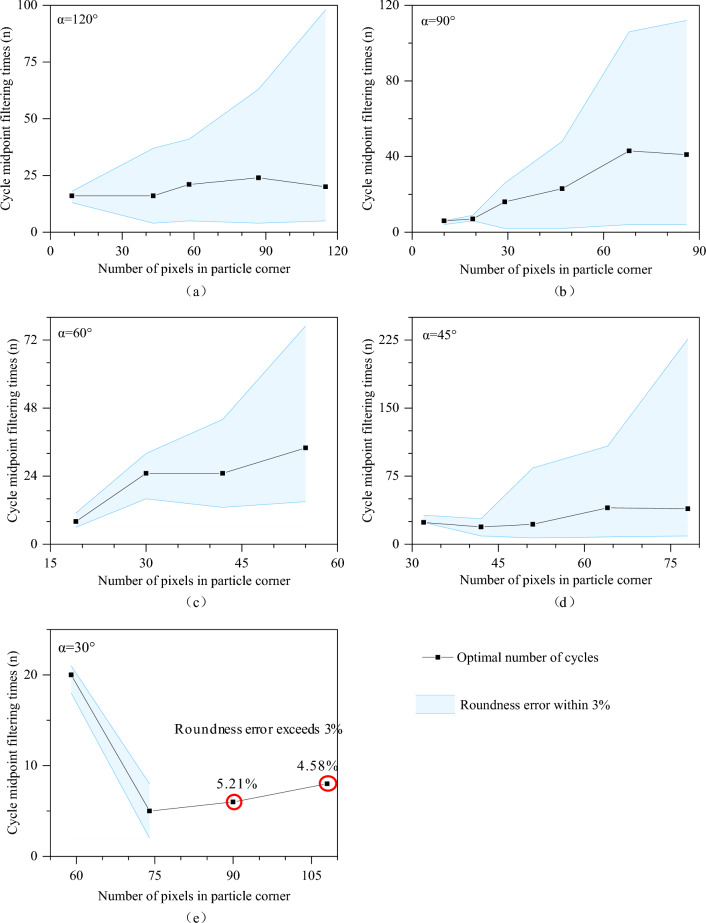
Relationship between the number of pixels at the corner (*m*) and the optimal range of cyclic filtering (*n*) operations under different *α* conditions.

**Figure 9:**
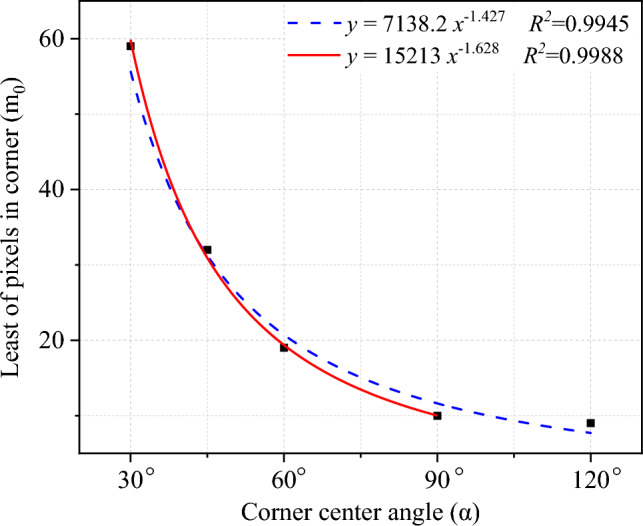
Relationship between *α* and the minimum number of pixels in the corner (*m*_*0*_). (*Note* The blue dashed line fitting curve includes all the data points, while the red solid line fitting curve does not include the data points corresponding to *α* = 120°. The red fitting curve fits better when *α* is small. Considering that the fitting curve is designed to estimate *m*_*0*_ at 12° < *α* < 30°, the fitting result achieved in red is used as the *m*_*0*_ estimation function).

We present the novel cyclic midpoint filtering method designed specifically for corner dealiasing. The outline extracted from the image has a certain degree of distortion, and the method proposed in this paper can restore its true state as much as possible when *α* ≥ 30°. This section establishes a connection between *m*, *α* and *n* and strictly controls the degree of dealiasing processing. This paper designs regular graphs with *α* values of 120°, 90°, 60°, 45°, and 30°, as shown in Fig. 7. According to the calculation results in Table 2, the relationship between *m* and *n*_*0*_ under different conditions of *α* is obtained, as shown in Fig. 8, which can ensure that corner deformation is reduced after dealiasing. An appropriate range of cyclic filtering numbers is obtained based on *α* and *m*. The interpolation method can be used to determine the values of the nonspecial angles mentioned above. By controlling the error of 2D Wadell roundness grinding within 3% to control the deformation of the corners, this section obtains the relationship between *α* and the minimum number of corner pixels (*m*_*0*_), as shown in Fig. 9. Several conclusions could be drawn from the results.

1. As *α* is held constant, the more pixels there are in the corner area (*m*), the wider the optional range of the cycle midpoint filtering times (*n*).
2. The larger *α* is, the lower the dependence on the number of pixels in the corner area (*m*) is, and the less sensitive the result is to a low number of pixels.

On the premise that *m* is sufficient to identify corners, when *α* ≥ 45°, the maximum calculation error does not exceed 3%. When *α* = 30°, the maximum error is 5.21%, as shown in Fig. 8e.

When *α* < 30°, the error gradually increases. The problem of an increase in the error caused by *α* < 30° can be alleviated by increasing the number of pixels at the corner (*m*) through interpolation. If *α* is known, according to the red curve fitted in Fig. 9, it is possible to estimate its corresponding *m*_*0*_. The number of pixels in the corner is increased to the corresponding *m*_*0*_ through interpolation, and then, dealifying processing is performed to balance smoothness and nondeformation, achieving the goal of reducing errors.

However, there are several limitations worth noting. As *α* gradually decreases, the proportion of key points dispersed to the circumference of the corner circle decreases, which also means fitting a large circle with a short arc, resulting in an increasing error during restoration. There is no clear value for how much *α* is not suitable for viewing as a particle corner. Based on our experience in the calculation process, setting *α* < 12° does not correspond to the particle corner. On the other hand, *α* < 12° does not correspond to the corner, which is set for the rationality of the definition of Wadell roundness corner.

### Efficiency of cyclic midpoint filtering

This research used Python for programming. When calculating the efficiency of the algorithm, the computer used is an ordinary laptop. The CPU used was an AMD Ryzen 7 (5700 U), the main frequency was 1.80 GHz, and the maximum altitude was 4.2 GHz.

**Figure 10:**
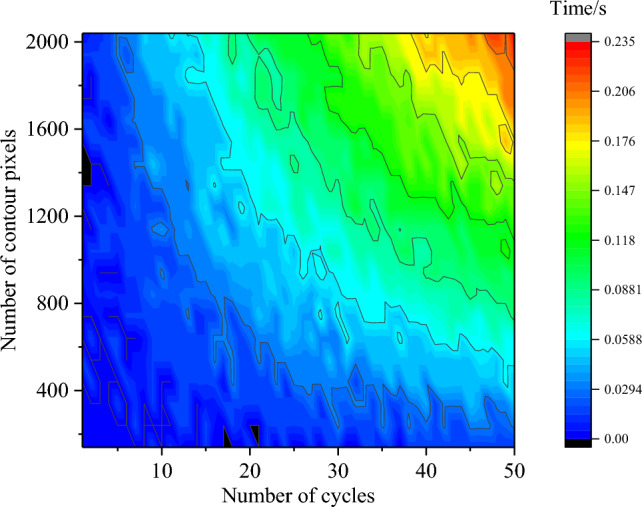
The time consumed by the cyclic midpoint filtering method.

The number of outline pixels and the number of cyclic filtering operations jointly determine the time required for noise reduction when a particle outline is denoised using the cyclic midpoint filtering method. This section discusses the time consumed for particles with a number of outline pixels in the range of 140–2040 and the corresponding number of cyclic filters, as shown in Fig. 10. The greater the number of outline pixels is, the greater the number of cyclic filtering operations, and the more time consumed. However, even if a particle outline composed of 2040 pixels is faced and 50 cyclic filtering and noise reduction operations are performed, the time is only 0.235 s. When the number of particle outline pixels is on this order of magnitude, the number of cyclic filtering operations will still not exceed 50.

In this study, 2D Wadell roundness calculations for a single particle executed at effective resolutions of 640 × 807 and 1400 × 1468 resulted in computation times of approximately 1.16 s and 5.49 s, respectively. At these two resolutions, the times required for the cyclic midpoint filtering method are 0.05 s and 0.12 s, accounting for approximately 4.3% and 2.1%, respectively, of the total calculation time for roundness. The higher the resolution is, the lower the proportion of time spent on dealiasing.

### Feasibility of extending to 3D Wadell roundness

The application of the proposed cyclic midpoint filtering method in dealiasing 3D particle surfaces. If the 3D surface of the particles is obtained through CT scanning, then it is certainly feasible. This is because the data obtained from CT scanning consists of a series of regular slice data, and the methods used to process individual slice data are the same as those used in this study. The calibration results of the dealiasing method proposed in this study can be directly applied to process slice data, and then restored to 3D contours to achieve dealiasing. If the point cloud data comes from 3D laser scanning, the cyclic midpoint filtering method proposed in this study may no longer be applicable. This is because point cloud data obtained in this way is typically disorderly in spatial distribution, and the use of the cyclic midpoint filtering method depends on strict planes, otherwise it will result in significant errors. However, the method of cycling can still be borrowed. Point cloud data with random distribution can be dealiased using weighted averaging methods, but the relationship between the range of weighted averaging and the dealiasing effect is difficult to establish. Perhaps a relatively fixed relationship can be found through small-scale weighted averaging, followed by iterative cycling.

The proposed key point grouping strategy for identifying particle corners is applicable in 3D surfaces. The strategy involves continuously searching for maximum spacing among ungrouped edge key points and setting breakpoints. However, when applied to 3D particle surfaces, Euclidean distance is used for evaluation. For example, clustering analysis can be performed using the k-Means algorithm, with k set to 2. Compared to directly applying adaptive clustering to distinguish particle corners, the method of continuous binary partitioning can reduce the interference of outliers on grouping. This approach is suitable for data obtained from CT and 3D laser scanning.

## Conclusions

1. The cyclic midpoint filtering method proposed in this paper for particle corner recognition can effectively address the problem of dense outline pixel aliasing. The larger *m* is, the wider the optimal range interval of *n* is. The larger *α* is, the lower its dependence on *m*. This method can smooth the particle outline while reducing deformation and distortion at the corners.
2. The strategy of grouping key points by combining dichotomy and cyclic methods can more effectively identify the corners of particles. It was found that the maximum spacing between the key points of the corners is most likely to serve as the boundary.
3. The method used in this paper calculates the 2D Wadell roundness of regular shapes, with an overall error of less than 3% and a maximum error of 5.21%. The roundness of the Krumbein chart particles calculated in this paper is also consistent with the reference range.

The study's findings have practical implications for dealing with dealiasing and conducting shape analysis on intricate closed contours commonly encountered in various industries. Our research results provide a reference for the quantification of 2D particle shape and indicate that digital image processing technology seems to be able to conveniently calculate other shape description indicators with quantitative significance. However, there are several limitations worth noting. Although our conclusion is supported by the 2D data, further research is needed in 3D cases. Therefore, future work should include studying the correlation between 2D and 3D particles, viewing cross-sectional CT scans of 3D particles as assemblies of a series of 2D images, and extending the 2D particle shape algorithm to 3D.

## Data Availability

Data is provided within the manuscript or supplementary information files.

## List of symbols

*α*: Central angle of the corner
*Π*: Value of 2D Wadell roundness
*m*: Number of corner pixels
*m*_*0*_: Minimum number of corner pixels within the allowable range of error
*N*: Number of corners of a particle
*n*: Number of cycles for dealiasing degree
*n*_*0*_: Optimal number of cycles for dealiasing degree
*R*: Maximum inscribed circle radius of a particle
*r*_*i*_: Curvature radius of the ith corner of a particle
